# Automated screening of potential organ donors using a temporal machine learning model

**DOI:** 10.1038/s41598-023-35270-w

**Published:** 2023-05-25

**Authors:** Nicolas Sauthier, Rima Bouchakri, François Martin Carrier, Michaël Sauthier, Louis-Antoine Mullie, Héloïse Cardinal, Marie-Chantal Fortin, Nadia Lahrichi, Michaël Chassé

**Affiliations:** 1 Centre Hospitalier de l’Université de Montréal, Montreal, Canada; 2 Centre Hospitalier Universitaire Sainte-Justine, Montreal, Canada; 3 Polytechnique Montréal, Montreal, Canada

**Keywords:** Diagnosis, Brain injuries, Machine learning, Organ transplantation

## Abstract

Organ donation is not meeting demand, and yet 30–60% of potential donors are potentially not identified. Current systems rely on manual identification and referral to an Organ Donation Organization (ODO). We hypothesized that developing an automated screening system based on machine learning could reduce the proportion of missed potentially eligible organ donors. Using routine clinical data and laboratory time-series, we retrospectively developed and tested a neural network model to automatically identify potential organ donors. We first trained a convolutive autoencoder that learned from the longitudinal changes of over 100 types of laboratory results. We then added a deep neural network classifier. This model was compared to a simpler logistic regression model. We observed an AUROC of 0.966 (CI 0.949–0.981) for the neural network and 0.940 (0.908–0.969) for the logistic regression model. At a prespecified cutoff, sensitivity and specificity were similar between both models at 84% and 93%. Accuracy of the neural network model was robust across donor subgroups and remained stable in a prospective simulation, while the logistic regression model performance declined when applied to rarer subgroups and in the prospective simulation. Our findings support using machine learning models to help with the identification of potential organ donors using routinely collected clinical and laboratory data.

## Introduction

Despite a modest growth in the number of organ donors in Canada over the last 20 years^1^, demand still exceeds supply for transplantable organs. In 2021^2^, while 4043 patients were on waiting lists, only 2782 organs were transplanted. That same year, 250 patients died while waiting for an organ.

Organ transplantation is critically dependent on potential organ donor identification and conversion to actual donors. The former is a major challenge that relies heavily on the training of medical teams, an inefficient approach given the rarity of deceased organ donation, especially in small centers^3^. Multiple retrospective cohort studies suggested that between 30 and 60% of potential organ donors are either not identified or not referred to an Organ Donation Organization (ODO)^4–7^. More efficient identification of potential organ donors could lead to an increase in the total number of referrals to an ODO and, therefore, to a potential increase in the number of organ donors.

Concurrently, there is a surge in the availability of healthcare data stemming from the rapid development and implementation of electronic health records (EHRs) and the interconnection of previously disjointed clinical databases. Advances in machine learning (ML) have shown great promise in making use of big data to improve health outcomes^8^. Neural networks (NN), a type of biologically inspired ML model, are capable of feats such as autonomous driving, image recognition, and pattern analysis. The application of NN models in medicine has been met with success^9^, including in the areas of medical imaging, and outcome prediction, such as mortality, and readmission^10^, and real-time complication prediction^11^.

The evolution of patients toward neurological death and potential candidacy for organ donation is a very complex clinical pattern. It remains unclear how ML approaches, such as NNs, would perform in such situations compared to simpler or more classical models^12^. More complex models could potentially be capable of detecting subtle patterns that are missed by simpler approaches, thereby identifying potential organ donors with greater accuracy. Most of the current applications of machine learning in the field of organ transplantation have focused on predicting recipients’ outcomes. Published models have suggested an improvement in the ability to predict survival of patients on organ transplantation waiting lists^13^, as well as survival of organ recipients^13–15^ and risk of graft rejection^16^ after successful transplantation. Other models have suggested the potential to help clinicians in selecting anti-rejection drug regimens after renal transplantation^17^. With respect to organ donor identification, a clinical score has been developed to estimate the probability of successful donation after cardiac death once a potential organ donor has been identified^18^. This score may be complementary to any predictive model identifying potential organ donors, but the latter has never been developed.

The primary objective of this study was to develop a predictive model for the identification of potential organ donors among patients admitted to an intensive care unit (ICU) using routinely collected clinical data. Our secondary objectives were: (1) to compare the discriminative property of a NN model compared to a logistic regression model used as a baseline, (2) to evaluate our models in prespecified subgroups of organ donor, and (3) to evaluate the model in a prospective simulation performed over a 48-h time.

## Methods

### Design, population, and outcome

This article follows the Transparent Reporting of a multivariable prediction model for Individual Prognosis or Diagnosis (TRIPOD) checklist^19^ and the Guidance for Development and Reporting of Prediction Models^20^. This study was approved by the Centre Hospitalier de l’Université de Montréal (CHUM) Research Ethics Board, that waived the requirement for individual informed consents given the low risk and retrospective nature of the study. All experiments and data treatments were performed in accordance with guidelines and regulations concerning retrospective private identifying information.

This study was based on a cohort of patients admitted to an ICU at the CHUM from January 1st, 2012 to December 31st, 2019, which had a minimal hospital length of stay of 16 h. For patients with multiple ICU admissions, we included data only for the latest ICU stay to avoid handling correlated data. Data were collected retrospectively from EHR data.

The predicted outcome was becoming a potential organ donor. Potential organ donors were defined as patients belonging to one of the four subgroups: (1) actual organ donor locally identified (admitted to the CHUM for a condition that eventually evolved to death and organ retrieval); (2) actual organ donor with neurological death diagnosis made in another hospital and transferred to the CHUM for organ retrieval; (3) potential organ donors referred to the ODO for donation but deemed ineligible for donation (substituted decision maker refusal, medical contraindications detected in the workup, etc.); (4) potential organ donors not referred to ODO. This last category of patients was identified by the ODO through local continuous death audits, which excluded patients with any recent, active, or metastatic cancer, disseminated infection, or multi-organ failure. The death audit defined potential organ donors as either a patient with a severe neurological condition, mechanically ventilated, who died within 24 h of the end of care or as a patient without a severe neurological condition, mechanically ventilated, who died within 3 h of the end of care.

### Predictors

#### Variable selection

Predictors included mainly time series of laboratory analyses and static clinical variables routinely collected as part of ICU care. We used only two static variables: the medical specialty responsible for the patient (neurosurgery, internal medicine, cardiology, etc.) and the presence or absence of a head radiological imaging exam.

Since patients identified as potential organ donors are cared for differently than other patients (e.g. more frequent investigations of certain types, absence of other types of investigations), we implemented measures to avoid learning from differences in medical practice^20^ stemming from evaluation for organ donation, rather than true clinical patterns. First, we did not include demographic and anthropomorphic variables (e.g. age, biological sex, height, or weight) as predictors, as they are not a priori exclusion criteria for becoming a potential organ donor. Second, we excluded rare laboratory analyses (defined as being ordered in less than 10% of all ICU patients) from the dataset. Finally, to mitigate the impact of an increased frequency of laboratory analyses often performed on potential organ donors, we only kept the last 72 h of a patient's stay (defined as ending through ICU discharge or death) divided into 9 blocks of 8 h, keeping only the last value within each time block. The final list of laboratory variables used in the model is reported in table S1 (supplementary material).

#### Missing values

The pattern of data missingness was assessed to likely be partially missing at random (since associated with the outcome of interest) and partially missing not at random (since clinicians tend to avoid ordering a laboratory test likely to be normal)^21^. Missingness for the medical specialty was handled as a distinct category. To mimic a clinician thinking about missing laboratory values, and to increase the model usability in a real-world setting, temporal data was imputed in a two-step method. First, the last value carried forward (LVCF) method was applied such that the value of a laboratory analysis performed in the previous 8-h block was carried forward in all subsequent blocks until updated by a new result. For values still missing after LVCF, we imputed values that were randomly sampled from an arbitrarily chosen Gaussian distribution. The mean and variance were chosen so its distribution contains 95% of the physiological normal range of each laboratory analysis. The normal range for each laboratory analysis was provided by the laboratory. This decision was made to reflect the fact that the decision by a clinician not to order a laboratory test likely reflects the hypothesis that the value is similar to the previous value or is expected to be in the normal range, while adding a variability to the imputed values.

### Model development

#### Model structure

We developed two models using different analytical approaches: a neural network temporal model (NN) and a logistic model (LM).

The NN model used the temporal aspect of the laboratory data combined with the static values. Its architecture is schematized in Fig S1 and Fig S2 (supplementary materials). We used a convolutive autoencoder (AE) to extract lower dimensionality features from time series of laboratory data^22,23^. AE is a subtype of NN architecture used in unsupervised learning. An AE is trained with the same information (image, text, laboratory data, etc.) presented at the entrance and at the exit of the network. The data is compressed and transformed into a representation of reduced size and dimensionality, called the latent representation, and is then decoded back to its original form. The AE learns to encode the data while minimizing loss of information. This type of architecture has been shown to be useful in multiple ways such as an embedding and dimensionality reduction tool, to reduce noise^24^, for transfer learning and pretraining^25^ and as an anomaly detection approach. We designed the convolutive AE based on ResNet^26^, a well-known convolutional neural network (CNN)^27^ using Python (version 3.7.4) and Keras (version 2.2.4) with TensorFlow (version 1.14)^28–30^. We adapted it to slide only along the temporal dimension of laboratory data, and to detect patterns in the temporal evolution of the laboratory values. We used dropout layers and L2 regularization on our models to help avoid overfitting. We used our AE to (1) take the maximum out of the temporal component of the data, (2) embed the temporal data in a one-dimensional format, (3) act as an anomaly detector since it was trained only on non-donor patients, and (4) function as a pretraining for a smaller size classifier. Our AE was only trained with the temporal data on a cohort of only non-donor patients. For the classifier model, we used a deep NN consisting of four fully connected layers ending with a sigmoid activation layer. The number of layers was chosen to balance capacity and complexity. Temporal embedded data were concatenated with static clinical data. Static data were encoded either as binary data or target mean encoding with smoothing for multiclass data. The final architecture is presented in Fig. S1 and S2 (supplementary material).

The logistic model (LM) used is a lighter baseline comparison model, using only the last laboratory value prior to ICU discharge or death concatenated with the static values, with a sigmoid activation layer. This model was significantly lighter in term of complexity of the prediction algorithm, but also in terms of number of data, since it only included the last time point of the time series.

#### Data structure

Potential organ donors are a rare subpopulation, encompassing around 2% of ICU patients based on preliminary data exploration. We approached the class imbalance problem with a mixture of purposeful subsampling and oversampling^31^. 85% of the non-donor patients were randomly selected and used as the embedding training set. It allowed subsampling while using this data for the autoencoder that did not require the outcome to develop the latent representation. The rest of the patients (15% of non-donors and all potential donors) were randomly divided into a train/validation/test set (60%/20%/20%). During training, proportionally more weight was put on the minority class. That means that if non-donors outnumber donors 100 to 2, the training weights of donors will be 100/2 = 50 and non-donors 1. The training and validation sets were used to develop the model, while the test set was excluded from the model development process. After a final model was developed, it was trained on both the train and validation datasets and results were estimated on the test set.

### Statistical analysis

The final model was trained using the combined train and validation sets, and the results were estimated using the test dataset. We reported performance, discrimination, and calibration properties of our models (NN and LM). We compared the overall performance of our NN model to our LM model using a scaled Brier score, their discriminative properties using AUROC and their calibration using calibration curves^20^. Confidence intervals were obtained using non-parametric bootstrapped percentiles (using 2000 resamplings of the test set). AUROC and Brier score from different models were compared using a Z statistic with a bootstrapped-based standard error (2000 resamplings) developed for paired models^32^.

In an approach to maximize potential donor detection, the goal was to choose a threshold with a high sensitivity. Since this study might have a potential prospective application, the optimal cutoff had to be derived from the training data and not from the test data. The cutoff was derived using a threefold cross-validation approach performed on the training dataset choosing the average threshold that gave a 90% sensitivity.

We performed two subgroups analyses. First, we compared the AUROC curve of our four subgroups by computing discrimination in each subgroup compared to the non-donor population. Second, we simulated a prospective approach to compare accuracy 48 h, 24 h and 8 h before ICU discharge. That was done by using only datapoints available progressively further from discharge. Finally, we did two sensitivity analyses. First, to ensure the model was resilient to removal of rare laboratory values, we iteratively retrained the models after progressively removing predictors. We started with predictor present in less than 10% all ICU patients, then 20%, then 30%, and so on. By doing so, the model was trained on fewer laboratory types at each step, using progressively only the most frequent ones. We analyzed the model performance at each step in a bootstrap approach. Second, we manually reviewed the files of patients not reported as potential organ donors by the death audit, but who were predicted as such with a high degree of confidence (predicted probability of 75% or more) by either model. We described qualitatively this data to adjudicate their real outcome in case the manual audit missed any patient and to help better understand the potential systematic bias and error patterns of the models. Statistical analyses were done using R (version 4.1.3) and Python (version 3.7.4)^29,33^.

## Results

Baseline characteristics of the population are reported in Table 1. Our complete dataset used 19 067 patients and included 397 potential donors. The prevalence of the outcome of interest was 2.1% in the study population, and 12% in the sub-sample used to train, validate and test of our models. After excluding rare laboratory analyses, the NN model and LM were trained on 103 distinct laboratory analyses, reported in the table S1 (supplementary material), as well as the two static predictors (the medical specialty responsible for the patient admission and the ordering of any head scan).

**Table 1:** Population characteristics.

	AE train n = 16,213	Training n = 1972	Validation n = 634	Testing n = 644
Sex (female) n(%)	5871 (36.2%)	737 (37.4%)	249 (39.3%)	262 (40.7%)
Age mean (std)	67.2 (14.5)	66.4 (14.7)	67.2 (14.2)	65.4 (15.6)
**Donors’ subtypes n (%)**				
Non-donor	16.213 (100%)	1734 (87.9%)	555 (87.5%)	564 (87.6%)
Transferred donors	–	121 (6.1%)	32 (5.0%)	36 (5.6%)
Local donors	–	53 (2.7%)	25 (3.9%)	29 (4.5%)
Referred but ineligible	–	42 (2.1%)	13 (2.1%)	10 (1.6%)
Potential not referred	–	22 (1.1%)	9 (1.4%)	5 (0.8%)
**Principals' reasons for admission n (%)**				
Ischemic heart diseases	3855 (22.9%)	375 (18.4%)	132 (20.8%)	130 (19.5%)
Other forms of heart disease	1833 (10.9%)	235 (11.5%)	60 (9.5%)	55 (8.2%)
Cerebrovascular diseases	1033 (6.1%)	175 (8.6%)	66 (10.4%)	59 (8.8%)
Diseases of the arteries/arterioles/capillaries	677 (4.0%)	75 (3.7%)	22 (3.5%)	18 (2.7%)
* Total Diseases of the circulatory system*	7907 (47.0%)	906 (44.5%)	306 (47.0%)	282 (42.3%)
Neoplasms	2729 (16.2%)	305 (15.0%)	104 (16.0%)	95 (14.2%)
Diseases of the digestive system	1649 (9.8%)	192 (9.4%)	49 (7.5%)	76 (11.4%)
Consequences of external causes	1299 (7.7%)	141 (6.9%)	54 (8.3%)	53 (7.9%)
Diseases of the respiratory system	1063 (6.3%)	132 (6.5%)	37 (5.7%)	43 (6.4%)
**Most frequent admission services n (%)**				
Cardiac surgery	5997 (37.0%)	615 (31.2%)	210 (33.1%)	199 (30.9%)
General surgery	1616 (10.0%)	184 (9.3%)	58 (9.1%)	57 (8.9%)
Hepatology	787 (4.9%)	121 (6.1%)	31 (4.9%)	33 (5.1%)
Internal Medicine	692 (4.3%)	107 (5.4%)	33 (5.2%)	34 (5.3%)
Neurosurgery	660 (4.1%)	99 (5.0%)	37 (5.8%)	34 (5.3%)
Intensive care	519 (3.2%)	176 (8.9%)	54 (8.5%)	52 (8.1%)
Burn unit	587 (3.6%)	64 (3.2%)	19 (3.0%)	27 (4.2%)
Length of stay in hours median [IQR]	50.7 [95.2]	51.6 [93.6]	51.4 [78.3]	48.5 [90.4]
Patients with any brain imaging n (%)	3381(20.9%)	538 (27.3%)	181 (28.5%)	133 (26.7%)
Death in ICU n (%)	1573 (9.7%)	413 (20.9%)	136 (21.5%)	133 (20.7%)

*AE train* auto-encoder training group; used only for the training of the unsupervised network.

**Figure 1:**
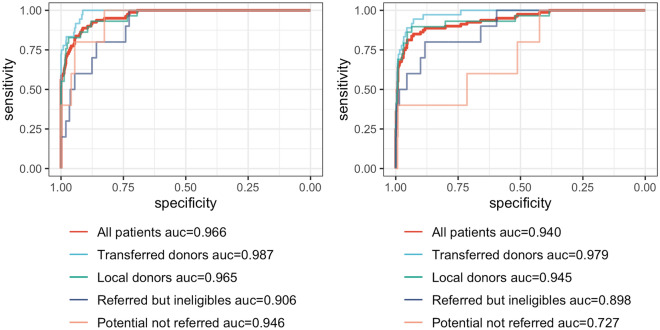
ROC Curves for all patients and subgroups of potential organ donors for the neural network (left) and the logistic model (right).

Overall, in the test dataset, the AUROC of the NN model (0.966; 95% CI 0.949–0.981) was marginally superior to the LM model (0.940; 95% CI 0.908–0.969); this difference was statistically significant (*p* = 0.014). The scaled Brier score was also statistically superior in the NN model (0.481 vs. 0.352, *p* = 0.049, Table 2). AUROC curves for each model as a whole and separated by organ donor subtypes are presented in Fig. 1.

**Table 2:** Models’ performance in the test set.

	NN model	Logistic model	*p* value
ROC AUC	0.966 (0.949–0.981)	0.940 (0.908–0.969)	0.014
Scaled Brier score	0.481 (0.306–0.614)	0.352 (0.135–0.518)	0.049
Sensitivity*	0.838 (0.750–0.914)	0.838 (0.750–0.917)	0.99
Specificity*	0.926 (0.903–0.947)	0.934 (0.913–0.954)	0.36

Data presented as bootstrapped median with 95% confidence interval. *p* values were calculated based on bootstrapped data. *at the cutoff estimated on the training set to target a sensitivity of 90%.

The cutoff, obtained by the threefold cross-validation approach described in the methodology, was 0.4 for the NN model and 0.47 for the LM model. Both models had similar sensitivities (84%) and specificity (93%) (Table 2). There was a difference between the aimed sensitivity (90%) and the obtained one (84% for both NN and LM, see Table 3). Confusion matrices are presented in Table 3. Confusion matrices by subgroups are presented in tables S2 to S6 (supplementary materials). To obtain an actual 90% sensitivity, the actual cutoff on the test set was 0.21 for the NN model and 0.08 for the LM. The confusion matrix at those cutoffs is presented in the Table S7 (supplementary materials). At those cutoffs, specificity was 88% for the NN model and 74% for the LM.

**Table 3:** Confusion matrix in the test set.

	True potential organ donors	True non-potential organ donors
**Neural network**		
Predicted potential organ donors	67	42
Predicted non-potential organ donors	13	522
**Logistic model**		
Predicted potential organ donors	67	37
Predicted non-potential organ donors	13	527

Calibration curves show that both models tend to underestimate the actual proportion of potential organ donors especially between a predicted probability of 0.3 and 0.8 with better accuracy at low and high predicted probabilities (see Fig S3, supplementary materials).

**Figure 2:**
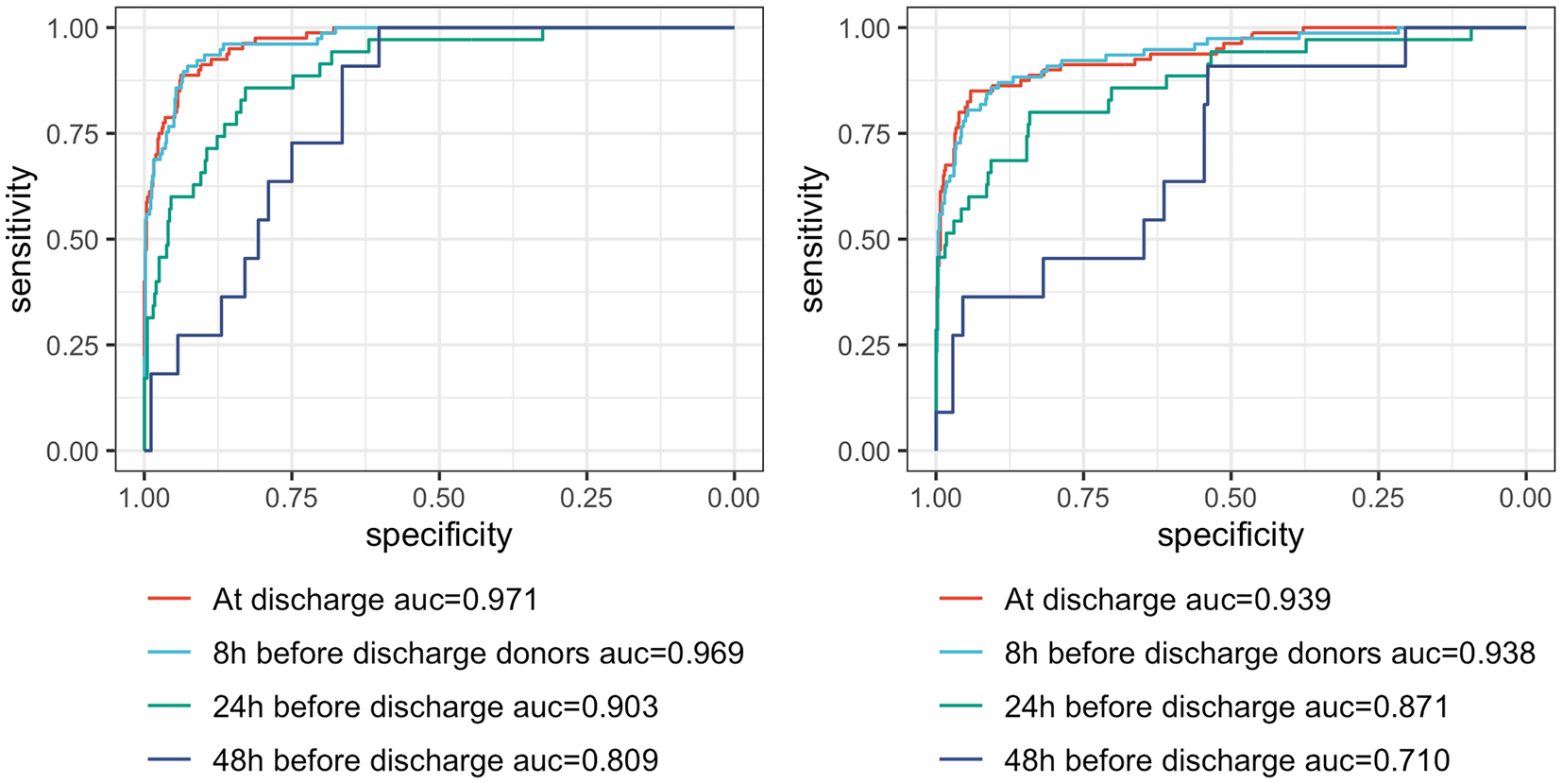
Simulation of a prospective analysis over 48 h before ICU discharge. ROC curve at each time point for the neural network (left) and the logistic model (right).

As a prespecified sensitivity analysis, we manually reviewed the medical files of the 11 patients who died in the ICU but were not identified as potential donors by our death audit, while predicted as donor by either model with a predicted probability of 75% or more. Results are presented in Table S8 (supplementary materials). Of 11 cases, almost half were potential donors who were excluded because of neoplasia. Two (# 10 and #11) could still have been referred because of the low likelihood of metastasis. From the non-neoplasia patients, two (#3 and #8) were actual potential organ donors missed by the death audit of our ODO. Sensitivity analysis showed that the model was resilient to laboratory removal, with only a small decrease in the AUROC when only the most frequent laboratory analyses were kept (Fig S4, supplementary materials). The simulated prospective approach (Fig. 2) showed that model AUROC decreased with longer delays between the ICU discharge and the test point. However, the NN keeps a better accuracy in the two longest delays of 24 and 48 h.

## Discussion

We proposed an innovative approach to the problem of organ donor identification. We were able to develop and internally validate a NN model to detect potential organ donors based on routinely collected clinical and laboratory data. We used EHR data with minimal pre-processing and minimal human intervention. We focused our effort on laboratory analyses, which are impartial, standardized, and accessible electronically, even in hospitals without EHRs.

This model is the first evidence supporting the use of real-world data to help screen for potential organ donors. To our knowledge, there is only one related model published^34^. This model was designed to identify catastrophic neurologic events using specific keyword identification on head CT scan reports. Given that most organ donors suffered a catastrophic neurologic event, this model could potentially be utilized to identify potential organ donors. It reported 77% sensitivity and 66% specificity. This approach required the scans to be interpreted, dictated, and transcribed by a radiologist, and thus required human intervention.

The more complex temporal model using NN marginally outperformed the non-temporal simpler version (LM). We compared the models with a bootstrap approach on a separate test set, instead of a cross-validation. Even if both approaches are similar in performance^35^, this enabled us to estimate the distribution of the results and apply statistical testing. However, it reduced the amount of data on which both models were compared and doesn’t replace an external validation dataset. The NN seemed to keep good discrimination in patients with more complex clinical patterns. It seemed to outperform the simpler LM when simulating a prospective identification of donors up to 48 h before the time of final donor classification. This could be explained by the added value of the clinical temporal evolution and by the fact that the NN had access to an embedded temporal vector of multiple laboratory data points, while the LM model only had access to the laboratory data measured at the latest time point before the outcome. Nonetheless, further work is needed to improve the model and to reduce the false positive rate. Since a lot of the false positives of the model were not eligible because of known neoplasia, that information could be in the future be used to update the models and improve their performance.

In subgroup analyses, we observed that our NN model performed better on donors that were also identified by the clinicians. These subpopulations represent the largest donor subtype making it likely that the model learned mostly from this subtype. Also, those subtypes may be more clinically distinct with more stable laboratory values, requested because of their donor status, making them easier to detect. Our model was also able to detect a significant proportion of potential organ donors that were missed or not referred by the clinical teams. Although the performance of the model was slightly lower in this group, our findings are of significant clinical interest, since those patients were missed by clinicians and did not have the opportunity to be assessed for donation. Since the detection of even one additional patient is of clinical benefit, we believe that if externally validated, such models could help support clinicians in the screening of potential organ donors. Interestingly, when we conducted a review of the classification errors of the models, the NN model detected two patients that have been missed by both the manual death audit and by the clinicians, potentially suggesting a higher sensitivity than the manual death audit alone.

Our study has a few limitations. First, our design required that a proportion of the data be used to train the autoencoder, reducing the amount of data available for the development of the classifier. Our approach has other advantages such as the possibility of easily merging multimedia information in future iterations of the model (radiology images, CT scans, vital signs, etc.). In addition, the design of our LM did not model the laboratory data evolution and restricted the absolute number of data point available, limiting comparisons of models and possibly explaining the slightly smaller discriminative property of the LM model. However, such limitation does not alter the absolute accuracy we measured of either model. Second, it is a retrospective study based on the data of a single quaternary transplant center, where clinicians are highly trained in the detection of potential organ donors. Truly missed organ donors are rare. In most of the non-referred patients, we found that organ donation was considered by the clinician and the option was not pursued, often because of family refusal. However, those patients still have a clinical pattern resembling a truly missed potential organ donor. It is unknown how the accuracy of our model would translate in a true, unsimulated, prospective setting, or in a different institution, and will thus require external and prospective validation before being considered for clinical use. Finally, our model requires at least 16 h of temporal data, and as such, does not apply to patients with catastrophic neurological events in whom a rapid decision would be required on a shorter time frame (e.g. in the emergency room). A decision for those patients will still need to be made by the clinician. Alternatively, those patients could benefit from an observation period in the ICU for better neurologic prognostication, as recommended by some experts, where our model would apply^36,37^.

In conclusion, we demonstrated the performance of two models identifying potential organ donors leveraging routinely collected clinical data. The more temporal NN model demonstrated slightly better and more stable performance. The models identified some patients that were not detected by the medical teams and manual death audits. Further work is required to validate the models externally and prospectively, and to further improve their prediction accuracy.

## Supplementary Information

Supplementary Information.

## Data Availability

Access to the data is restricted to the research team by the ethical board according to provincial laws but may be made available for audit purposes after privacy assessments and appropriate legal agreements. For information, please contact Dr. Michaël Chassé, senior author of this work. Code for the NN and the LM model are available on the team’s github at https://github.com/compass-network/clinical.
