# Supplementary material for: Automated screening of potential organ donors using a temporal machine learning model
Source: Sci Rep. 2023 May 25;13:8459. doi: 10.1038/s41598-023-35270-w (PMC10212939; doi:10.1038/s41598-023-35270-w)
Supplement: Supplementary file 1 — Supplementary Information. [file 41598_2023_35270_MOESM1_ESM.docx]

# Supplementary material

1. Neural Network architecture


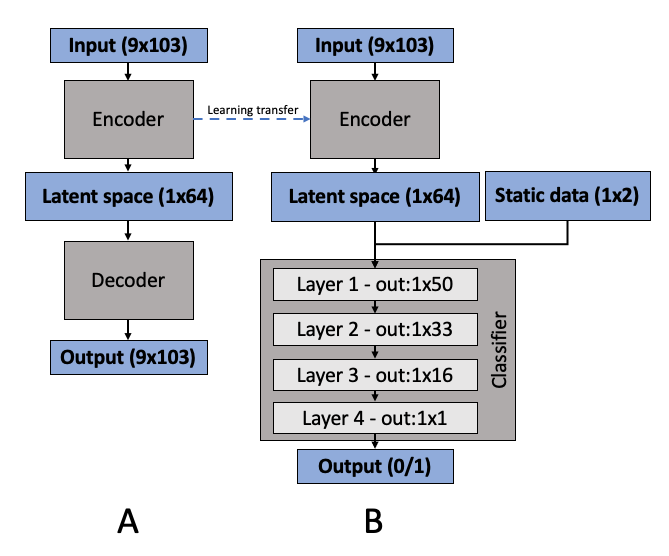


A) Autoencoder. B) Classifier

1. AutoEncoder architecture


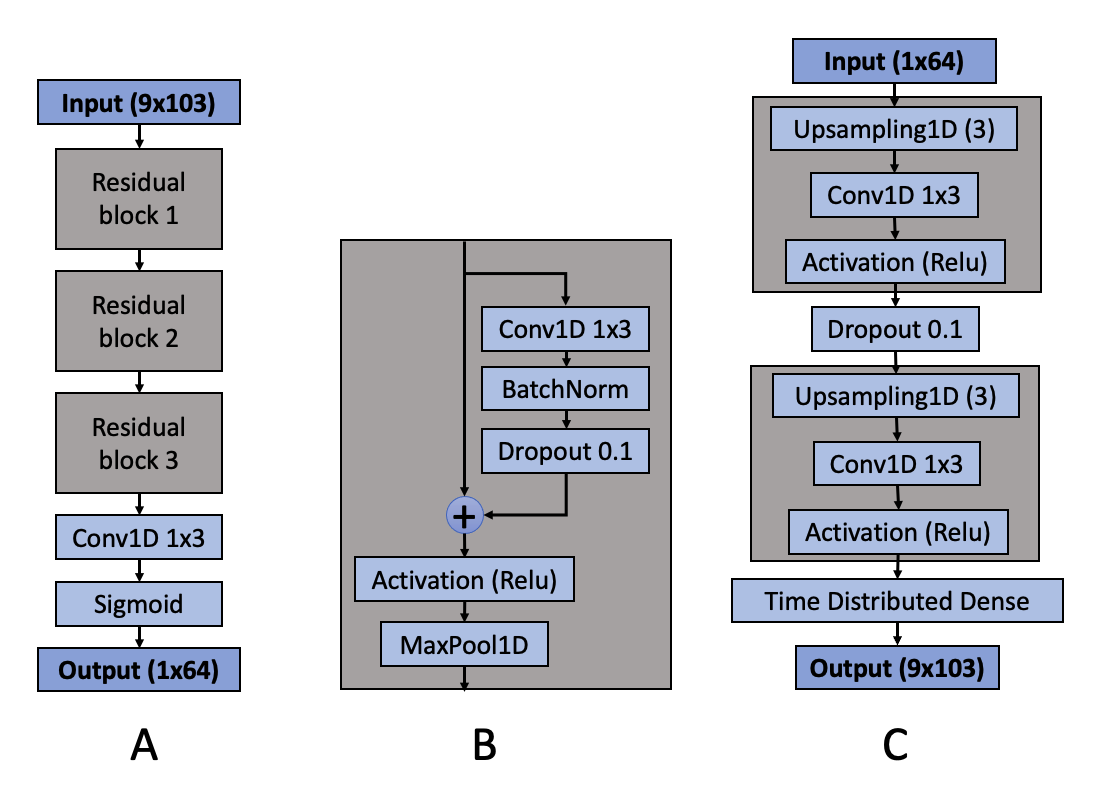


A) Encoder. B) Residual block C) Decoder. Technical acronyms refer to the types of layers used in the Keras API. For more information please see the API documentation at <https://keras.io/api/layers/>)

1. Laboratory values used in model development

| Lab Value | %missing | Median | IQR | Normal range |
| --- | --- | --- | --- | --- |
| white_blood_cell_count | 0,1% | 10,5 | 6,7 | [4-11] |
| hemoglobin | 0,1% | 96,0 | 32,0 | [120-196] |
| hematocrit | 0,1% | 0,3 | 0,1 | [0,35-0,6] |
| red_blood_cell_deviation_width | 0,1% | 14,8 | 2,7 | [11,5-20] |
| red_blood_cell_count | 0,1% | 3,2 | 1,0 | [3,8-6,2] |
| platelet_count | 0,1% | 188,0 | 129,0 | [140-500] |
| mean_corpuscular_volume | 0,1% | 90,9 | 6,8 | [80-101] |
| mean_corpuscular_hemoglobin_concentration | 0,1% | 331,0 | 18,0 | [300-365] |
| mean_corpuscular_hemoglobin | 0,1% | 30,2 | 2,5 | [24-33,5] |
| lipemia_presence | 0,1% | 0,0 | 0,0 | [0-0] |
| icterus_presence | 0,1% | 0,0 | 0,0 | [0-0] |
| hemolysis | 0,1% | 0,0 | 0,0 | [0-0] |
| Neu­­hil_count | 0,1% | 8,0 | 6,3 | [1,3-7,7] |
| monocyte_count | 0,1% | 0,8 | 0,6 | [0-1,6] |
| lymphocyte_count | 0,2% | 1,1 | 1,0 | [1-4,1] |
| sodium | 0,2% | 140,0 | 5,0 | [135-145] |
| creatinine | 0,2% | 81,0 | 59,0 | [42-112] |
| potassium | 0,2% | 4,0 | 0,7 | [3,5-5] |
| eosinophil_count | 0,3% | 0,1 | 0,2 | [0-0,8] |
| basophil_count | 0,4% | 0,0 | 0,0 | [0-0,3] |
| urea | 0,4% | 7,2 | 6,2 | [2,8-8,8] |
| mean_platelet_volume | 0,7% | 10,3 | 1,4 | [6,5-13,5] |
| glucose | 1,1% | 7,5 | 3,1 | [4-6,2] |
| chloride | 1,2% | 106,0 | 7,0 | [96-106] |
| magnesium | 1,2% | 0,8 | 0,2 | [0,7-1,01] |
| phosphate | 1,4% | 1,1 | 0,4 | [0,72-1,64] |
| albumin | 2,3% | 28,0 | 9,0 | [36-52] |
| total_calcium | 2,7% | 2,1 | 0,3 | [2,17-2,56] |
| inr | 3,6% | 1,1 | 0,3 | [0,8-1,2] |
| partial_thromboplastin_time | 3,8% | 29,0 | 18,0 | [22-32] |
| creatine_kinase | 14,2% | 195,0 | 397,0 | [24-213] |
| total_bilirubin | 15,0% | 13,0 | 14,3 | [7-23] |
| alanine_aminotransferase | 15,0% | 30,0 | 53,0 | [8-39] |
| aspartate_aminotransferase | 15,7% | 39,0 | 57,0 | [13-39] |
| hs_troponin_t | 16,1% | 112,0 | 333,8 | [0-18] |
| corrected_total_calcium | 23,6% | 2,3 | 0,2 | [2,2-2,58] |
| alkaline_phosphatase | 24,1% | 75,0 | 65,0 | [36-110] |
| venous_ph | 25,8% | 7,4 | 0,1 | [7,31-7,43] |
| fio2 | 27,2% | 0,4 | 0,2 | [0,21-0,21] |
| venous_pco2 | 29,1% | 44,0 | 11,0 | [38-54] |
| venous_bicarbonate | 29,1% | 24,0 | 5,2 | [21-29] |
| venous_po2 | 29,1% | 39,0 | 11,0 | [35-95] |
| arterial_po2 | 29,2% | 108,0 | 57,6 | [70-110] |
| arterial_pco2 | 29,2% | 38,0 | 10,0 | [32-45] |
| arterial_bicarbonate | 29,2% | 23,0 | 4,6 | [19-28] |
| venous_lactic_acid | 33,1% | 1,3 | 1,0 | [0,56-2,4] |
| venous_o2_sat | 33,6% | 0,7 | 0,2 | [0,7-1] |
| lipase | 34,0% | 23,0 | 29,0 | [10-102] |
| arterial_o2_sat | 34,2% | 1,0 | 0,0 | [0,92-1] |
| total_protein | 37,2% | 56,0 | 14,0 | [63-81] |
| urinary_ph | 38,3% | 5,0 | 1,5 | [4,8-8] |
| urinary_density | 38,4% | 1,0 | 0,0 | [1-1,03] |
| urinary_protein | 38,4% | 0,0 | 0,3 | [0-0] |
| urinary_glucose | 38,5% | 0,0 | 0,0 | [0-0] |
| urinary_blood | 38,5% | 0,3 | 25,0 | [0-0] |
| urinary_bilirubin | 38,5% | 0,0 | 0,0 | [0-0] |
| urinary_cetones | 38,6% | 0,0 | 0,0 | [0-0] |
| urinary_urobilinogen | 38,7% | 0,0 | 0,0 | [0-0] |
| urinary_nitrite | 38,9% | 0,0 | 0,0 | [0-0] |
| urinary_leucocytes | 39,0% | 0,0 | 0,0 | [0-0] |
| ionized_calcium_ph74 | 42,0% | 1,2 | 0,1 | [1,12-1,32] |
| arterial_ph | 44,0% | 7,4 | 0,1 | [7,35-7,45] |
| lactate_dehydrogenase | 45,6% | 205,0 | 142,0 | [104-205] |
| fibrinogen | 47,8% | 3,4 | 2,2 | [2-4,5] |
| anticoagulant | 48,4% | 0,0 | 1,0 | [0-0] |
| gamma_glutamyl_transferase | 48,9% | 44,0 | 75,0 | [7-47] |
| amylase | 51,5% | 55,0 | 60,5 | [20-104] |
| temperature | 53,8% | 37,0 | 0,0 | [36-38] |
| osmolality | 55,3% | 289,0 | 16,0 | [275-300] |
| urinary_polychromia | 55,4% | 1,0 | 0,0 | [0-0] |
| thrombin_time | 56,2% | 17,0 | 7,0 | [12-18] |
| ph | 58,6% | 7,4 | 0,1 | [7,37-7,43] |
| nucleated_red_blood_cells | 60,2% | 0,0 | 0,0 | [0-0,1] |
| erythrocytes | 63,9% | 4,0 | 54,0 | [0-2] |
| ck_mb | 65,0% | 16,0 | 24,3 | [0-19] |
| leucocytes_count | 65,3% | 4,0 | 7,0 | [0-2] |
| uric_acid | 67,4% | 310,0 | 178,0 | [167-441] |
| arterial_lactic_acid | 67,9% | 1,4 | 1,4 | [0,6-2,4] |
| anisocytosis_presence | 68,2% | 1,0 | 1,0 | [0-0] |
| base_excess | 68,8% | -1,4 | 5,2 | [-2,5-2,5] |
| anion_gap | 71,2% | 8,0 | 4,0 | [4-14] |
| venous_base_excess | 71,6% | -0,7 | 6,0 | [-2-3] |
| cholesterol | 72,3% | 3,4 | 1,6 | [3,16-7,3] |
| triglycerides | 72,7% | 1,4 | 1,0 | [0,43-2,82] |
| hdl_cholesterol | 74,3% | 0,9 | 0,4 | [0,8-2,38] |
| plt_anisocytosis_presence | 75,3% | 1,0 | 0,0 | [0-0] |
| urinary_mucus_presence | 75,6% | 1,0 | 0,0 | [0-0] |
| urinary_bacteria | 76,1% | 1,0 | 0,0 | [0-0] |
| elliptocytes_presence | 77,6% | 1,0 | 0,0 | [0-0] |
| urinary_pavimentous_cells_presence | 77,9% | 1,0 | 0,0 | [0-0] |
| echinocyts_presence | 81,4% | 1,0 | 0,0 | [0-0] |
| thyroid_stimulating_hormone | 82,0% | 2,1 | 2,6 | [0,35-5,5] |
| direct_bilirubin | 83,9% | 19,6 | 29,6 | [0-3,6] |
| urinary_ac_ascorb | 84,1% | 0,0 | 0,0 | [0-0] |
| giant_platelets_presence | 84,4% | 1,0 | 0,0 | [0-0] |
| hba1c | 85,2% | 0,1 | 0,0 | [0,04-0,06] |
| acanthocytes_presence | 85,9% | 1,0 | 0,0 | [0-0] |
| urinary_hyalin_cylinder_presence | 86,3% | 1,0 | 3,0 | [0-0] |
| atypia_lympho_presence | 87,6% | 1,0 | 0,0 | [0-0] |
| globulins | 87,8% | 28,0 | 11,0 | [21-34] |
| toxic_granulation_presence | 87,8% | 1,0 | 0,0 | [0-0] |
| target_cells_presence | 88,1% | 1,0 | 0,0 | [0-0] |
| doehle_body_presence | 88,3% | 1,0 | 0,0 | [0-0] |

Values are presented in median and interquartile range (IQR). Colors are made to reflect the groupings used in supp. Fig S4.

1. Confusion matrix in test set – Transferred donors

| Neural Network | | |  |
| --- | --- | --- | --- |
|  | True potential organ donors | True non-potential organ donors |  |
| Predicted potential organ donors | 33 | 42 |  |
| Predicted non-potential organ donors | 3 | 522 |  |
|  | | | |
| Logistic model | | | |
|  | True potential organ donors | True non-potential organ donors |  |
| Predicted potential organ donors | 33 | 37 |  |
| Predicted non-potential organ donors | 3 | 527 |  |

1. Confusion matrix in test set – Local donors

| Neural Network | | |  |
| --- | --- | --- | --- |
|  | True potential organ donors | True non-potential organ donors |  |
| Predicted potential organ donors | 24 | 42 |  |
| Predicted non-potential organ donors | 5 | 522 |  |
|  | | | |
| Logistic model | | | |
|  | True potential organ donors | True non-potential organ donors |  |
| Predicted potential organ donors | 26 | 37 |  |
| Predicted non-potential organ donors | 3 | 527 |  |

1. Confusion matrix in test set – Referred but ineligibles

| Neural Network | | |  |
| --- | --- | --- | --- |
|  | True potential organ donors | True non-potential organ donors |  |
| Predicted potential organ donors | 6 | 42 |  |
| Predicted non-potential organ donors | 4 | 522 |  |
|  | | | |
| Logistic model | | | |
|  | True potential organ donors | True non-potential organ donors |  |
| Predicted potential organ donors | 6 | 37 |  |
| Predicted non-potential organ donors | 4 | 527 |  |

1. Confusion matrix in test set – Potential not referred

| Neural Network | | |  |
| --- | --- | --- | --- |
|  | True potential organ donors | True non-potential organ donors |  |
| Predicted potential organ donors | 4 | 42 |  |
| Predicted non-potential organ donors | 1 | 522 |  |
|  | | | |
| Logistic model | | | |
|  | True potential organ donors | True non-potential organ donors |  |
| Predicted potential organ donors | 2 | 37 |  |
| Predicted non-potential organ donors | 3 | 527 |  |

1. Confusion matrix in validation set – All potential donors

| Neural Network | | |  |
| --- | --- | --- | --- |
|  | True potential organ donors | True non-potential organ donors |  |
| Predicted potential organ donors | 68 | 36 |  |
| Predicted non-potential organ donors | 11 | 519 |  |
|  | | | |
| Logistic model | | | |
|  | True potential organ donors | True non-potential organ donors |  |
| Predicted potential organ donors | 72 | 46 |  |
| Predicted non-potential organ donors | 7 | 509 |  |

1. Confusion matrix in test set – All donors – Actual sensitivity 90%

| Neural Network | | |
| --- | --- | --- |
|  | True potential organ donors | True non-potential organ donors |
| Predicted potential organ donors | 72 | 67 |
| Predicted non-potential organ donors | 8 | 496 |
|  | | |
| Logistic model | | |
|  | True potential organ donors | True non-potential organ donors |
| Predicted potential organ donors | 72 | 144 |
| Predicted non-potential organ donors | 8 | 420 |

1. Calibration plot


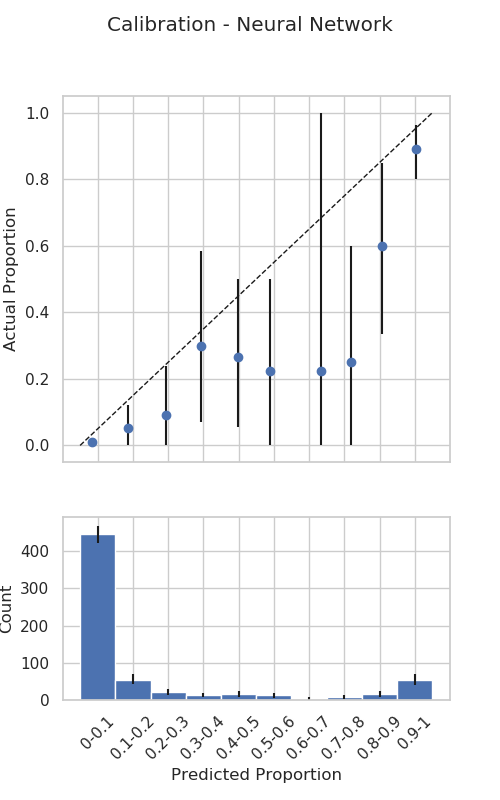

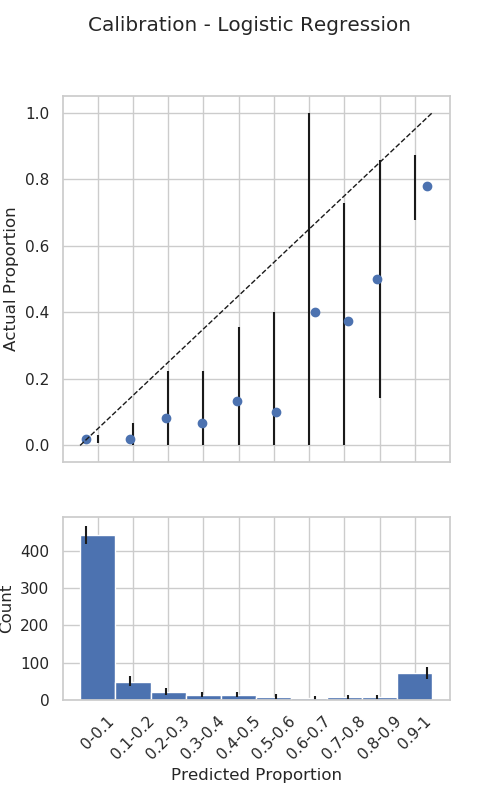


1. False positive error checking

| Pt | NN score | LM score | Clinical history |
| --- | --- | --- | --- |
| 1 | 0.79 | 0.45 | Male over 80 years old. Pneumonia with small need of supplement oxygen. Past medical history of strokes. Withdrawal of care despite clinical improvement of pneumonia. |
| 2 | 0.40 | 1.00 | Male between 60 and 70 years old. Pneumonia post pneumonectomy for lung neoplasia. |
| 3 | 0.89 | 0.59 | Male between 60 and 70 years old. Hypertensive intracerebral hemorrhage. Death within 24h. Not referred because of "Heavy history of cardiac and pulmonary problems" |
| 4 | 0.57 | 0.97 | Female over 80 years old. Admission for pulmonary septic shock. Withdrawal of care because of slow evolution |
| 5 | 0.90 | 0.27 | Female between 60 and 70 years old. Aspiration pneumonia. Past medical history of chronic pulmonary obstructive disease dependent on oxygen. Rapid progression to withdrawal of care despite improving labs. |
| 6 | 0.81 | 0.60 | Male between 70 and 80 years old. Pneumonia post craniectomy for intracerebral metastasis. |
| 7 | 0.55 | 1.00 | Male between 60 and 70 years old. Pneumonia post pneumonectomy for lung neoplasia. |
| 8 | 0.79 | 1.00 | Male between 50 and 60 years old. Intracerebral aneurysm rupture. Family refusal before ODO referral. |
| 9 | 0.26 | 0.95 | Male between 20 and 30 years old. Fulminant neurodegenerative disease. Withdrawal of care. |
| 10 | 0.84 | 0.12 | Male between 30 and 40 years old. Chondrosarcoma. Cardiac arrest with to brain ischemia. Withdrawal of care. |
| 11 | 0.50 | 0.99 | Male between 50 and 60 years old. Subarachnoid hemorrhage secondary to a biopsy of a glioblastoma. |

1. Sensitivity analysis


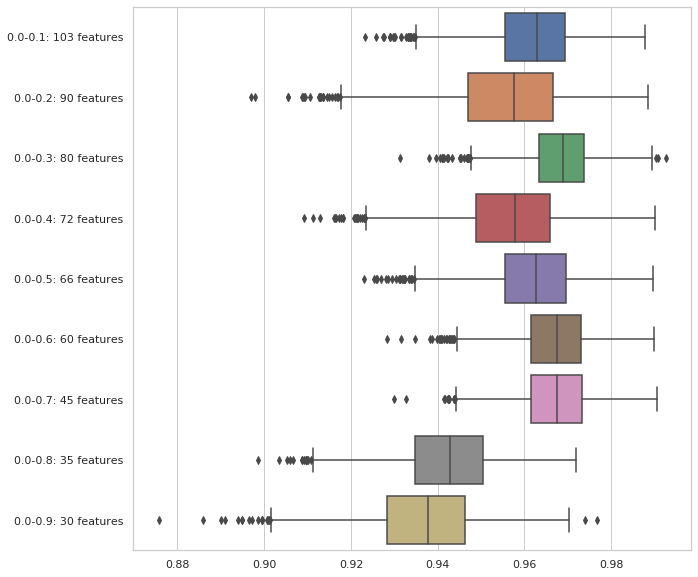


Boxplot of bootstrapped AUC (x-axis) with training with a progressively smaller number of predictors (y-axis). Predictors were removed based on missingness, the blue boxplot being all predictors present in at least 10% of patients and the bottom beige boxplot representing predictors present in at least 90% of patients.
